# Levels of islet amyloid polypeptide in cerebrospinal fluid and plasma from patients with Alzheimer’s disease

**DOI:** 10.1371/journal.pone.0218561

**Published:** 2019-06-17

**Authors:** Nina Schultz, Shorena Janelidze, Elin Byman, Lennart Minthon, Katarina Nägga, Oskar Hansson, Malin Wennström

**Affiliations:** 1 Clinical Memory Research Unit, Department of Clinical Sciences Malmö, Lund University, Malmö, Sweden; 2 Department of Acute Internal Medicine and Geriatrics, Linköping University, Linköping, Sweden; 3 Memory Clinic, Skåne University Hospital, Malmö, Sweden; Nathan S Kline Institute, UNITED STATES

## Abstract

The biologically active pancreatic hormone peptide islet amyloid polypeptide (IAPP) regulates brain functions such as appetite and cognition. It also plays a role in clearance of amyloid beta (Aβ), a peptide implicated in the dementia disorder Alzheimer’s disease (AD). If IAPP becomes modified, it loses its biological activity and starts to aggregate. Such aggregations have been found in the AD brain and decreased plasma levels of the unmodified IAPP (uIAPP) have been shown in the same patients. In the current study, we analyze levels of uIAPP and total IAPP (unmodified and modified) in cerebrospinal fluid (CSF) to investigate its potential as a biomarker for AD. We found no differences in neither CSF nor plasma levels of uIAPP or total IAPP in AD patients compared to cognitive healthy individuals (NC). The levels of uIAPP in CSF of NC were positively correlated with uIAPP in plasma, Q-albumin and albumin levels in CSF, but negatively correlated with CSF levels of t-tau and p-tau. These findings were not seen in AD patients. Levels of total CSF IAPP correlated positively with total Q-albumin and albumin levels in CSF in both AD and NC. In addition, total plasma IAPP correlated positively with CSF t-tau and p-tau in NC and negatively with CSF Aβ_42_ in AD patients. To conclude, our studies did not find evidence supporting the use of CSF IAPP as an AD biomarker. However, our findings, indicating a compromised translocation of uIAPP in and out of the brain in AD patients as well as the correlations between total plasma IAPP and AD biomarkers, encourage further research on the role for IAPP in AD.

## Introduction

The neurodegenerative disorder Alzheimer’s disease (AD) is neuropathologically characterized by progressive accumulation of amyloid beta (Aβ)_42_ into senile plaques, as well as intraneuronal tangles, composing of hyperphosphorylated tau (p-tau). These hallmarks are in clinical routine monitored by analysis of cerebrospinal fluid (CSF), where reduced levels of Aβ_42_ and increased levels of p-tau and total tau (t-tau) are considered to reflect increased amount of trapped aggregated Aβ_42_ and neuronal damage/degeneration, respectively. The senile plaques and tangles are associated with neuronal cell death, neuroinflammation and atrophy in brain areas involving memory and other cognitive functions. However, vascular changes (including impaired blood-brain barrier (BBB) function, reduced vessel integrity, aberrant angiogenesis, reduced number of vessel-supporting pericytes and micro bleeds) are also commonly found in the affected brain areas [1–3]. Moreover, increased CSF levels of endothelial/pericyte markers (such as vascular endothelial growth factor (VEGF)[4], soluble platelet-derived growth factor receptor-β (sPDGFRβ) [5] as well as markers for BBB disruption (Q-albumin [6, 7] and fibrinogen [8])) have been seen in AD patients. These findings together with the known increased risk of AD in individuals with vascular diseases (such as type 2 diabetes (T2D) and cardiovascular disease [9, 10]), highlight vasculopathy as a contributing factor in AD pathology.

We and others have in recent studies demonstrated a potential link between vasculopathy and islet amyloid polypeptide (IAPP) [11–14]. This pancreatic β-cell derived peptide is co-secreted with insulin and while insulin is known to facilitate uptake of glucose from the blood, IAPP regulates the amount of glucose released into the bloodstream. Biologically active IAPP circulates in the blood and crosses the BBB into the brain, where it is known to regulate appetite by activating noradrenergic neurons in the area postrema (AP) [12, 15, 16]. Interestingly, IAPP has recently been suggested to also play a role in Aβ clearance, as studies have shown increased Aβ translocation from the brain into the blood after peripheral injections of IAPP or its non-amyloidogenic analogue pramlintide [17]. This protective role for IAPP against AD pathology is further demonstrated in a preclinical study, where transgenic AD mice improved cognitively after treatment with IAPP or pramlintide [17, 18]. A clinical study, demonstrating a positive correlation between plasma levels of IAPP and cognition [19], supports the idea of IAPP as being beneficial and further indicates that a loss of the same may be detrimental. Indeed, reduced levels of IAPP in plasma [18], retina and brain [20] of AD patients have been reported. The reduction may reflect either decreased production of IAPP or alterations in the IAPP structure. Studies favor the latter suggestion, as high concentrations and/or modifications (either by C-terminal deamidation or by reduction of the C2-C7 disulphide bond) of IAPP lead to loss of biological activity and formation of toxic aggregates. Such aggregates are known to form deposits in peripheral organs [14, 21] [22] of patients with type 2 diabetes (T2D), but have also recently been found in the brain of demented T2D patients and patients with AD [23]. The brain depositions of aggregated IAPP were found both in the parenchyma and within the vessel walls [13], where the latter indicates an association between aggregated IAPP and changes in the vasculature. This idea is further supported by a study where we, by the use of human brain tissue studies and cell culture studies, showed that oligomeric IAPP is internalized by pericytes and induces cell death [11]. The depositions of aggregated IAPP in the brain, inevitably raise the question whether such entrapment of IAPP can be reflected in the CSF, similarly to Aβ. To our knowledge, IAPP levels in CSF of AD patients have only been reported once previously. This study used an ELISA capturing foremost unmodified IAPP to demonstrate increased IAPP levels in AD patients compared to individuals with normal cognition [24]. The analyzed cohort size was however very small (AD n = 8 and NC n = 7) and the findings need to be verified in a larger cohort. In the current study, we use the same ELISA to verify whether levels of unmodified IAPP (hereon called uIAPP) in CSF are altered in a larger cohort of AD patients and moreover investigate if the CSF levels are associated with plasma levels of uIAPP, cognitive decline, AD biomarkers and Q-albumin (as a marker for BBB integrity). In addition, we use different antibodies directed against epitopes present in both modified and unmodified IAPP to, by the use of Western blot and an in-house-made direct ELISA, analyze the total amount of IAPP levels in CSF.

## Material and methods

### Individuals included in the study

The patient cohort consisted of cognitive healthy individuals (n = 44) and patients clinically diagnosed with AD (n = 37, whereof n = 7 were diagnosed with T2D). CSF and plasma samples were obtained at the Memory Clinic at Skåne University Hospital (Sweden). Both controls and AD patients underwent cognitive and neurological assessments by a physician with special interest in dementia disorders. Patients with AD were diagnosed according to DSM-IV Criteria for Alzheimer’s disease. The cognitive healthy individuals displayed no neurological or cognitive deficiency symptoms. Basic CSF AD biomarkers (Aβ_42_, p-Tau and t-Tau) were analyzed using Euroimmun ELISA (Euroimmun AG, Lübeck, Germany) and albumin levels in plasma and CSF were measured by immunoturbidimetry on a Roche Cobas Analyzer (Roche Diagnostics, Bromma, Sweden). The albumin ratio (Q-Alb) was calculated as CSF albumin (mg/l)/plasma albumin (g/l) and was used as a measure of BBB function. The responsible clinician determined whether the patient had decision making capacity before study enrollment. All individuals gave written informed consent to participate in the study. The study and the informed consent form were approved by the ethics committee in Lund, Sweden and all investigations were conducted according to the Declaration of Helsinki.

### Plasma and CSF sampling

Blood plasma and CSF samples were drawn between 8 AM and 12 AM with the patients non-fasting. CSF was collected in polypropylene tubes and mixed gently to avoid gradient effects. All samples were centrifuged within 30 minutes at +4°C, 2000 x *g* for 10 minutes to remove cells and debris. Samples were stored in aliquots at −80°C until used. The procedure followed the Alzheimer's Association Flow Chart for LP and CSF sample processing [25].

### Analysis of IAPP levels

Levels of unmodified IAPP in CSF and plasma were analyzed using Human Amylin ELISA kit (Millipore, Darmstadt, Germany) according to manufacturers’ instructions. CSF samples were analyzed undiluted and plasma samples were diluted 1:40. Levels of total IAPP in CSF and plasma were analyzed using an in-house developed direct ELISA according to previously published protocol [20]. CSF and plasma samples were pre-treated with 3M Guanine hydrochloride 2 h RT before analysis. CSF samples were diluted 1:160 and plasma samples 1:100 000 in PBS and analyzed in triplicates.

### Western blot analysis

The presence of IAPP in CSF were also analyzed using Western Blot, by loading CSF samples in 4x SDS sample buffer, separated by Tris-HCl gels (Bio-Rad Laboratories, Hercules, CA), transferred onto a PVDF membrane (Bio-Rad Laboratories) and boiled in PBS for 5 minutes in order to expose further epitopes and improve antibody binding [23]. Monomeric and pre-aggregated human IAPP control peptide (AlexoTech AB, Umeå, Sweden) was prepared according to previously published protocol [11] and used as positive controls. The membranes were incubated 1h RT in blocking solution (BS) (5% nonfat milk in PBS-Tween) before incubated with primary antibodies A133 directed towards hIAPP aa 20–29 (1:1000, kind gift Professor Gunilla Westermark, Uppsala University, Sweden) [23], T4149 directed towards C-terminal of hIAPP (1:1000, Peninsula Laboratories, San Carlos, CA) or T4157 directed towards hIAPP aa 25–37 (1:1000, Peninsula Laboratories) diluted in BS overnight at 4°C. Membranes were rinsed and incubated with ECL goat anti-rabbit secondary antibody (ab6721, 1:10000, Abcam, Cambridge, UK) 1 h RT before the proteins were visualized using chemiluminescence detection (Bio-Rad). The intensity of the protein bands in digitalized images of immunoblotted membranes was analyzed using Image Lab 4.0.1 (Bio-Rad Laboratories).

### Statistical analysis

Statistical analysis was performed using SPSS software (version 24.0 for Mac, SPSS Inc., Chicago, IL). Two CSF unmodified IAPP values and three plasma unmodified IAPP values were above 10 times the SD of the mean and therefore considered as outliers and excluded from the analysis. Normal distribution was analyzed using Kolmogorov-Smirnov test. Values not normally distributed were logarithmic transformed before analysis. Differences between patient groups were analyzed using ANOVA followed by Dunnett post-hoc test and student t-test. ANCOVA was used when co-variants were taken into considerations. Correlation analyses values by the use of Pearson correlation test. Results are presented as mean ± standard deviation. A p < 0.05 was considered as significant.

## Results

### Analysis of CSF and plasma unmodified IAPP levels

There were no significant differences in the logarithmically transformed uIAPP levels in either CSF or plasma between patients with AD, AD+T2D and NC (0.98 ± 0.35, 1.01 ± 0.64, 0.90 ± 0.45, p = 0.680 and 2.45 ± 0.21, 2.49 ± 0.39, 2.35 ± 0.22, p = 0.156, respectively). Also, similar results in CSF and plasma uIAPP between AD, AD+T2D and NC were found when age and gender were used as co-variates. Moreover, when uIAPP levels in plasma were used as co-variate, there were still no significant differences between patients with AD, AD+T2D and NC in levels of uIAPP in CSF (0.98 ± 0.35, 1.01 ± 0.65, 0.90 ± 0.46, p = 0.985). However, analysis using student t-test showed a trend towards a significant increase in uIAPP plasma levels in AD patients compared to NCs (p = 0.077).

### Correlations between CSF and plasma unmodified IAPP levels

**Fig 1:**
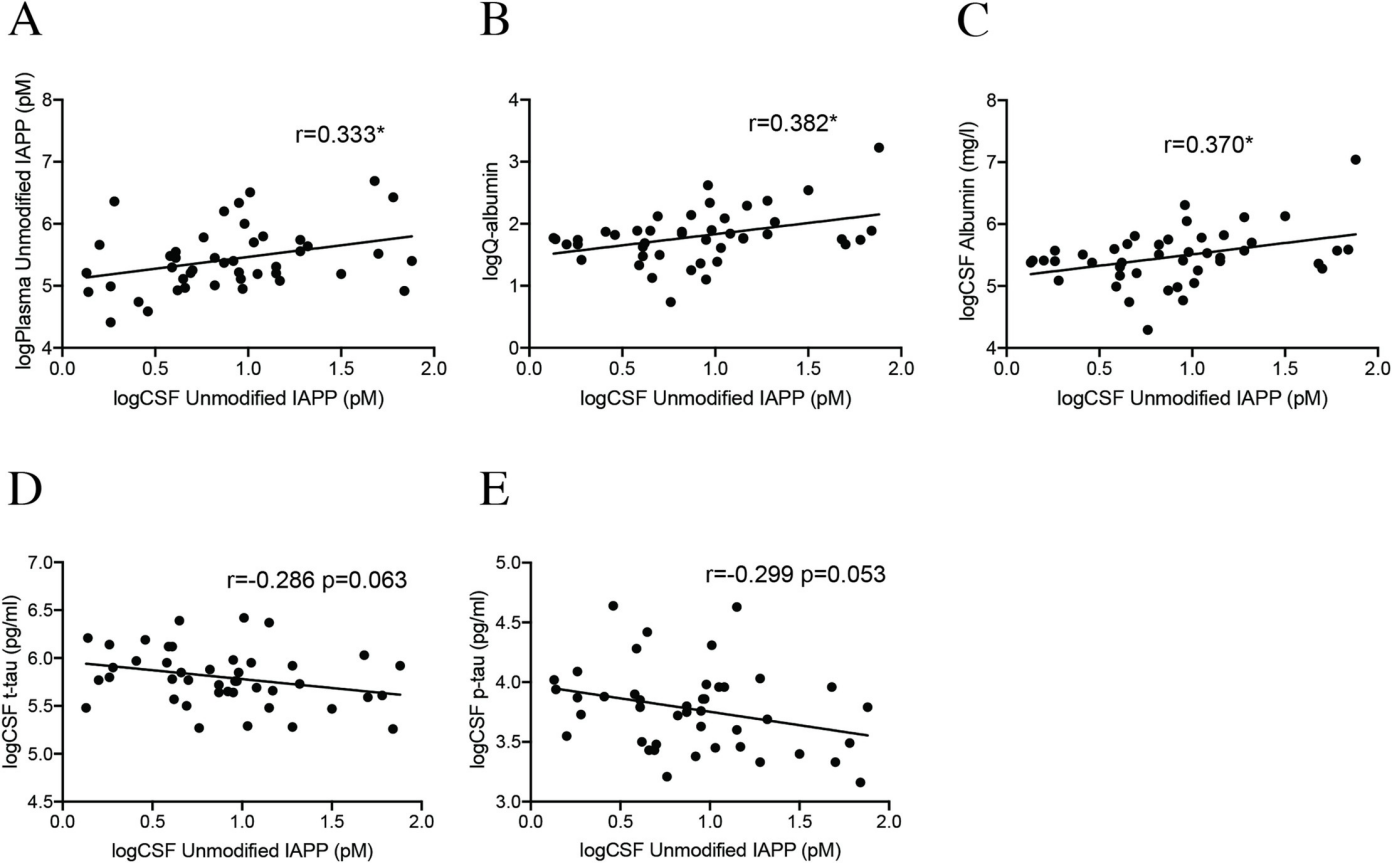
Correlations between CSF unmodified IAPP and Q-albumin, CSF albumin and AD biomarkers. Scatter plots demonstrating significant correlations between logarithmic transformed values of unmodified CSF IAPP and logarithmic transformed values of unmodified plasma IAPP (A), logarithmic transformed values of Q-Alb (B), logarithmic transformed values of CSF albumin levels (C), logarithmic transformed values of CSF t-tau levels (D) and logarithmic transformed values of CSF p-tau levels in cognitively healthy individuals (NCs)(E). Data was analyzed with Pearson correlation test. * Significant correlation at p<0.05 level.

Furthermore, our analysis showed that plasma and CSF uIAPP levels correlated positively across the group (r = 0.364, p = 0.001). This correlation remained significant in the NCs (Fig 1A), but was lost in the AD group and the AD+T2D group (S1A and S1B Fig, respectively) when the groups were analyzed separately. The CSF levels in AD and AD+T2D remained unaltered compared to NC when plasma levels of unmodified IAPP were included as a covariate (p = 0.899) and the ratio between plasma IAPP levels and CSF IAPP levels did not differ between AD patients, AD+T2D patients and NCs (p = 0.482). Individuals carrying one or two ApoE4 allele showed a tendency to significantly increased levels of plasma uIAPP compared to ApoE4 non-carrying individuals (2.35 ± 0.22, 2.44 ± 0.24, p = 0.075), but this tendency was not detected when the diagnosis groups were analyzed separately. Levels of uIAPP in CSF did not significantly differ in APOEε4 carriers compared to APOEε4 non-carriers, regardless of whether the analysis was performed across the groups or on the groups separately.

### Correlations between CSF levels of unmodified IAPP and Q-albumin

The ratio between albumin levels in plasma and CSF, i.e. the Q-albumin, did not differ between AD patients and NCs (Table 1, p = 0.680), but since CSF levels of IAPP correlated with plasma levels of IAPP, we found it interesting to analyze the association between CSF IAPP levels and Q-albumin. The CSF IAPP levels correlated with Q-albumin across the groups (r = 0.292, p = 0.008). The correlation remained significant in NCs (Fig 1B), but not in AD or AD+T2D group (S1C and S1D Fig), when the patient groups were analyzed separately. Finally, CSF levels of uIAPP and albumin correlated significantly in the NC group (Fig 1C), but not in the AD or AD+T2D patient group (S1E and S1F Fig, respectively).

**Table 1:** Data on individuals included in the study.

Variables	NC (n = 44)	AD (n = 30)	T2D + AD (n = 7)
Age (years)	73.41 ± 6.16	73.57 ± 6.56	75.57 ± 11.24
Gender (M/F)	13/31	9/21	1/6
APOEε4 carrier (no)	17	21	4
MMSE	29.18 ± 0.81	19.50 ± 4.26***	20.57 ± 5.13***
Q-albumin	6.68 ± 3.73	6.80 ± 2.54	8.39 ± 6.23
CSF albumin (mg/l)	267.20 ± 164.03	268.967 ± 108.28	311.14 ± 221.27
CSF Aβ_42_ (pg/ml)	790.76 ± 291.04	386.70 ± 110.29***	522.51 ± 112.73*
CSF t-tau (pg/ml)	344.34 ± 105.12	621.81 ± 207.42 ***	683.78 ± 213.30***
CSF p-tau (pg/ml)	46.11 ± 18.21	120.40 ± 41.23***	119.14 ± 57.43***
CSF uIAPP (pM)	2.74 ± 1.38	2.81 ± 0.88	3.20 ± 1.72
Plasma uIAPP (pM)	262.81 ± 162.86	316.82 ± 159.52	428.78 ± 162.41
CSF IAPP WB (abs)	289447.81± 89972.04^a^	262526.62 ± 72777.26^b^	246513.14 ± 80454.55
CSF total IAPP (abs)	1.36 ± 0.18	1.37 ± 0.16	1.36 ± 0.10
Plasma total IAPP (abs)	1.31 ± 0.17	1.31 ± 0.18	1.21 ± 0.23

NC = cognitive healthy individuals controls, AD = Alzheimer’s disease patients, AD+T2D = patients with Alzheimer’s disease and type 2 diabetes.

a = data is missing on NC n = 1.

b = data is missing on AD n = 1. Data is analyzed using ANOVA followed by Dunnett post-hoc test and values are presented as mean value ± SD.

* Significant correlation at p<0.05 level.

** Significant correlation at p<0.01 level.

*** Significant correlation at p<0.001 level.

### Correlations between CSF levels of unmodified IAPP and AD biomarkers

Correlation analysis across the groups did not reveal associations between CSF levels of uIAPP, t-tau, p-tau or Aβ_42_. However, when the patient groups were analyzed separately, a tendency to significant negative correlation was seen between uIAPP and t-tau as well as p-tau (Fig 1D and 1E) in the NC group. These correlations were not seen in the AD group (S1G and S1I Fig, respectively), but a tendency to significance (S1H and S1J Fig, respectively) was detected in the AD+T2D group. No correlations were found between MMSE scores, regardless whether the analysis was performed across the groups or on the groups separately.

### Analysis of CSF IAPP using Western blot

**Fig 2:**
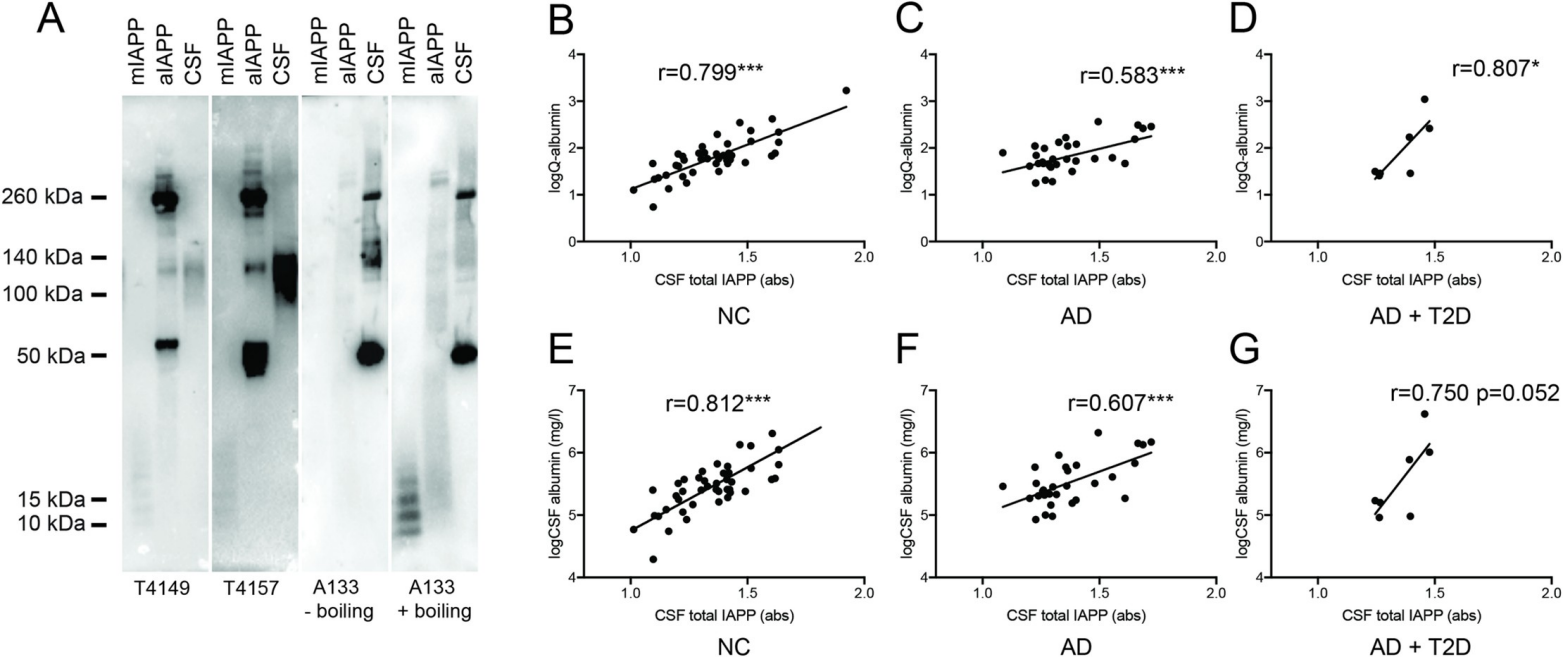
Western blot analysis of islet amyloid polypeptide (IAPP) preparations and cerebrospinal fluid (CSF) and correlations between total IAPP, Q-albumin, CSF albumin and AD biomarkers. Immunoblotting with T4149, T4157 recognized low molecular weight (A133,) IAPP in wells loaded with monomeric preparations of IAPP (mIAPP), whereas A133 only recognized the LMW after membrane boiling (A). All three antibodies also recognized several bands with various molecular weights (ranging from 10 kDa to over 300kDa) in the pre-aggregated preparation of IAPP (aIAPP) and a 120 kDa in CSF (A). The A133 detected additional bands in CSF both before and after membrane boiling (A). Scatter plots in (B-D) demonstrate correlations between total CSF IAPP and logarithmic transformed values of Q-albumin in NC (B), AD patients (C) and patients with both AD and T2D (D). Scatter plots in (E-G) demonstrate correlations between total CSF IAPP and logarithmic transformed values of CSF albumin in NC (E), AD patients (F) and patients with AD and T2D (G). Data was analyzed with Pearson correlation test. * Significant correlation at p< 0.05. ** Significant correlation at p<0.001. *** Significant correlation at p< 0.0001.

In order to study whether there are differences in composition (i.e. various aggregation forms of IAPP) in the CSF from AD, AD+T2D and NC, we analyzed the CSF samples also by the use of Western Blot. We used three different antibodies (T4149, T4157 and A133). Both T4149 and T4157 could detect low and high molecular weight (MW) IAPP in monomeric preparations and pre-aggregated preparations of synthetic control IAPP, respectively. The A133 antibody detected only high MW, but after boiling and re-staining the membrane also low MW IAPP was detected (Fig 2A). Similar treatment and re-staining of membrane with T4149 and T4157 yielded no bands, suggesting a destruction of the required epitope. All three antibodies recognized a band with the MW of approximately 120 kDa in CSF (Fig 2A), and additional strong bands were seen on the membrane boiled and re-stained with A133 (60 kDa, 140kDa, 260 kDa). No bands were found below 50 kDa. Analysis of the 120 kDa band showed no significant differences in intensity between patients with AD, AD+T2D and NC (Table 1, p = 0.258) and did not significantly differ in APOEε4 carriers compared to APOEε4 non-carriers or between genders. The intensity moreover did not correlate with any of the AD biomarkers (t-tau, p-tau or Aβ_42_) or MMSE regardless of whether the analysis was performed across the groups or on the groups separately. Two individuals in the AD+ T2D patient group and one in the NC group displayed a different band pattern compared to AD and the remaining NC and AD+T2D group (S2B Fig).

### Analysis of total CSF IAPP levels

Although we found no differences in the 120 kDa band intensities between groups, we found it important to investigate the intensity of all bands visualized by A133. We therefore analyzed levels of total CSF IAPP by the use of an in-house-developed direct ELISA with A133 as detection antibody. We found no significant differences between groups (Table 1), but levels of total CSF IAPP correlated positively with both Q-albumin and levels of CSF albumin across the group (r = 0.703, p = 0.000 and r = 0.720, p = 0.000, respectively). These correlations remained significant also when the patient groups were analyzed separately (Fig 2B–2G).

### Analysis of total plasma IAPP

**Fig 3:**
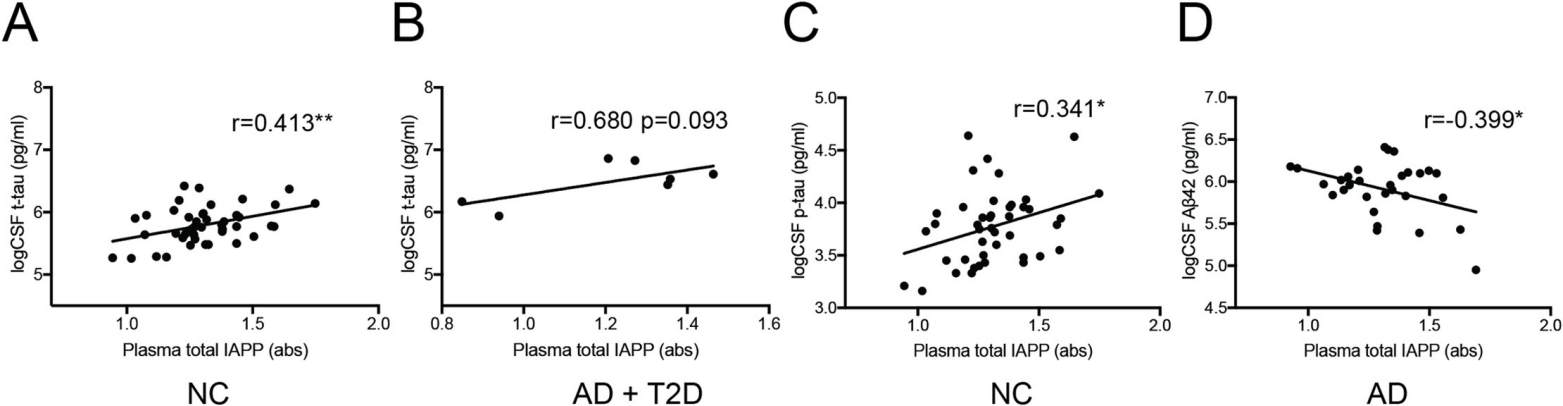
Correlations between total plasma IAPP and AD biomarkers. Scatter plots demonstrate correlation between levels of total plasma IAPP and logarithmic transformed values of CSF t-tau in NCs (A) and a trend towards a correlation in patients with AD+T2D (B). Scatters plots also demonstrate a positive correlation between levels of total plasma IAPP and logarithmic transformed values of CSF p-tau in NCs (C) as well as a negative correlation between levels of total plasma IAPP and logarithmic transformed values of CSF Aβ42 in AD patients (D). Data was analyzed using Pearson correlation test. * Significant correlation at p<0.05.

Finally, we also analyzed levels of total IAPP in plasma using our in-house developed ELISA. We found no significant differences between groups (Table 1) and no significant correlations between total plasma IAPP and total CSF IAPP, regardless of patient group. Furthermore, total plasma IAPP did not correlate with AD biomarkers across the groups, but when the groups were analyzed separately, positive correlations were found between total plasma IAPP and CSF t-tau in NCs (Fig 3A) as well as a trend towards a correlation in patients with AD and T2D (Fig 3B). We also found a positive correlation between total plasma IAPP and CSF p-tau in NCs (Fig 3C) as well as a negative correlation between total plasma IAPP levels and CSF Aβ_42_ in AD patients (Fig 3D).

## Discussion

In the current study, we use a commercially available ELISA to detect unmodified IAPP (uIAPP) in plasma and CSF of patients with AD, AD+T2D and NCs. The analysis revealed no significant alterations between the studied groups in regard to either plasma or CSF levels of uIAPP. These results are both in line with and contradict previously published studies using the same ELISA. Fawver et al. demonstrated in 2014 [24] unaltered plasma uIAPP levels in AD patients compared to healthy controls, whereas Adler et al. the same year instead showed reduced plasma levels of uIAPP in AD patients and patients with mild cognitive impairment (MCI) compared to cognitively intact individuals (n = 206 AD, 64 MCI and 111 NC) [18]. Fawver et al. further showed increased CSF levels of uIAPP in AD patients compared to healthy controls (AD n = 8 and NC n = 7) [24], a finding we were unable to replicate. These contradicting results should be viewed from the fact that measuring IAPP in plasma and CSF is accompanied by complications. Secretion of IAPP from the pancreas is regulated by blood glucose levels and thus IAPP levels fluctuate during the day in relation to food intake. Collection of CSF in clinical routine is generally performed without fasting prescriptions and day time restrictions and fasting have not been taken into consideration in either our study or the studies by Adler et al. and Fawver et al. In an attempt to compensate for the lack of fasting control, we also compared CSF uIAPP levels when plasma uIAPP levels were used as a covariate and the ratio between CSF and plasma levels of uIAPP. The analysis still showed no alterations between the groups, indicating that CSF uIAPP is not profoundly altered in AD patients. Despite these precautions we cannot completely rule out the possibility that uIAPP is altered in CSF of AD patients and conclude that further studies on larger cohorts with fasting prescriptions are warranted.

To further investigate if alterations in IAPP levels in AD patients can be found when both modified and unmodified IAPP are analyzed, we examined CSF levels using Western Blot analysis with three different antibodies (4149, 4157 and A133) directed against epitopes unaffected by modifications in the N- and C-terminal regions (i.e. both modified and unmodified IAPP). All three antibodies revealed a band at 120kDa, but the A133 antibody revealed several additional bands including strong bands at approximately 60 kDa and 260 kDa. Since pre-aggregated IAPP loaded on the same gel also yielded strong bands at the same positions (i.e. 60 kDa, 120 kDa and 260 kDa), it is tempting to speculate that IAPP preferably form aggregates with these molecular weights. Although analysis of the 120 kDa band indicated a decrease in intensity in AD patients, the difference was not significant compared to NC. Similar findings, i.e. unaltered levels in AD patients, were seen when we analyzed the CSF with our in-house developed direct ELISA based on the A133 antibody. We thus draw the conclusion that levels of total CSF IAPP, in similarity to uIAPP levels, are not profoundly altered in AD patients. This corresponds with our previous studies where we found no differences in levels of uIAPP or total IAPP in soluble fractions from hippocampal homogenates from AD patients compared to NCs [20].

The found correlation between plasma and CSF levels of uIAPP in the NC group suggests a well-functional translocation of peripherally-derived uIAPP over the BBB under normal conditions. This crossing is important for brain processes dependent on IAPP access, such as appetite regulation and cognition. Interestingly, the correlation between CSF and plasma uIAPP levels was lost in patients with AD both with and without T2D; a result indicating either that i) normal BBB crossing of uIAPP is compromised or ii) clearance of uIAPP into the CSF is dysfunctional in these patients. In contrast to uIAPP, levels of total IAPP in plasma did not correlate with the same in CSF. This disassociation could be due to a limitation of the amount of IAPP that can cross the BBB, but it is tempting to hypothesize that some of the IAPP, possibly the modified IAPP, gets stuck in the brain parenchyma and is not cleared into the CSF.

The found correlations between CSF levels of both uIAPP and total IAPP and the BBB intactness marker Q-albumin could further imply that entering of IAPP (regardless of modification form) into the brain is dependent on BBB permeability. Considering this idea, it is important to point out that several previous studies have shown altered BBB barrier function (demonstrated by analysis of different variables including Q-albumin [4, 26], CSF levels of fibrinogen as well as postmortem immunohistological stainings against BBB markers) in AD patients. The AD patients included in our cohort did not display increased Q-albumin compared to the NCs and we cannot therefore exclude the possibility that analysis on an AD patient group with increased Q-albumin would yield elevated levels of uIAPP in likeness to the study by Fawver et al. It should further be pointed out that the strong correlation between Q-albumin and total IAPP found in all groups also could indicate an impact of modified or aggregated IAPP on BBB intactness. Such hypothesis would fit in well with our previous study demonstrating a strong toxic impact of oligomeric IAPP on brain pericytes [27], cells helping to preserve BBB function [28, 29].

The results demonstrating that CSF albumin levels correlate with both CSF uIAPP and total CSF IAPP in NCs further indicate a potential role for albumin in the translocation of IAPP over the BBB. Albumin has been shown to assist the translocation of Aβ_42_ over the BBB [30] and since IAPP resembles Aβ in many ways [31], it is not unlikely that albumin plays a similar role in translocation of IAPP over the BBB and/or into CSF. Interestingly, the found correlations between uIAPP and albumin were not detected in CSF from AD or AD+T2D patients. This could mean that the albumin dependent translocation of uIAPP is compromised in these patients, an idea in line with findings demonstrating decreased Aβ-albumin complexes in blood of AD patients which were hypothesized to reflect a decreased ability of albumin to bind and remove Aβ from the brain, leading to an increased cerebral Aβ accumulation [32].

Our next step was to investigate whether cognitive decline or AD biomarkers were associated with uIAPP or total IAPP levels. Neither CSF uIAPP nor plasma uIAPP correlated with MMSE or Aβ levels, regardless of groups being analyzed, but we found a near to significant negative correlation between CSF uIAPP and t-tau as well as p-tau levels in NCs and a similar trend in the AD+T2D patient group. These results should be viewed from the perspective that increased t-tau and p-tau levels in CSF are indicative of neurodegeneration, and hence the results support the hypothesis that uIAPP is beneficial and protective, in this case against tau pathology. This finding is in line with a previous study demonstrating reduced aggregation and phosphorylation of tau in the brain of 5XFAD (a mouse model of AD) after IAPP treatment [33]. From this point of view, it was further interesting that we found a significant positive correlation between plasma levels of total IAPP (where harmful modified IAPP in addition to uIAPP were analyzed) and CSF levels of both t-tau and p-tau in NC. It is thus tempting to hypothesize that our results reflect the impact of peripherally-derived harmful IAPP on neuronal integrity, as hypothesized by previous researchers [12]. Similar reasoning can be applied on our found negative correlation between total plasma IAPP levels and CSF Aβ levels in AD patients, suggesting that increased amount of aggregation-prone IAPP deriving from the periphery could seed Aβ and thereby contribute to the accumulation of Aβ in the brain [12] (which lowered levels of Aβ levels in CSF are indicative of).

Finally, the cohort included 7 AD patients with T2D, a cohort too small to yield results wherefrom reliable conclusions can be drawn. Nevertheless, since modification and aggregation of IAPP are clinical features of T2D, one would expect changes visible even in such a small cohort. Since CSF and plasma uIAPP levels of AD+T2D patients were very similar to levels in AD and NCs, we draw the conclusion that T2D pathology in general does not add to any drastic changes in plasma or CSF uIAPP levels. It is however important to remember that T2D is a very heterogenous patient group and thus it may well be that alterations in IAPP plasma or CSF levels only are found in subgroups of T2D patients with specific IAPP pathology. Our Western blot analysis highlights this potential scenario as two AD + T2D patients showed a divergent band pattern (shown in S2 Fig).

To conclude, our study aimed to investigate whether levels of uIAPP and total IAPP in CSF and plasma are altered in AD patients and if these levels are associated with AD biomarkers, cognitive decline and BBB permeability. Since we found no alterations in uIAPP and total IAPP levels in CSF and plasma and unaltered intensity of the 120kDa IAPP band in CSF of AD patients, we conclude that our studies do not contribute with evidence supporting the use of IAPP as an AD biomarker. However, the lost associations between i) Q-albumin and CSF uIAPP levels ii) CSF uIAPP and plasma uIAPP levels in AD patients indicate that brain uptake of uIAPP or translocation of uIAPP into CSF are affected in AD. Moreover, since associations were also found between i) total CSF IAPP and Q-albumin ii) total plasma IAPP and AD biomarkers, we suggest that the modified IAPP in total IAPP causes increased BBB permeability, neurodegeneration as well as Aβ accumulation. Given these findings, we cannot rule out the potential role of IAPP in AD pathology and encourage further studies on the relation between IAPP and vascular/pericytes markers as well as studies on potential alterations of modified IAPP or total IAPP.

## Supporting information

S1 Fig Scatter plots demonstrating correlation analysis between CSF unmodified IAPP and Q-albumin, CSF albumin and AD biomarkers. Logarithmic transformed values (log) of CSF unmodified islet amyloid polypeptide (IAPP) where not significantly correlated with logPlasma unmodified IAPP in patients with Alzheimer's disease (AD) (A) and patients with AD and type 2 diabetes (AD+T2D) (B), logQ-albumin in AD patients (C) and patients with AD+T2D (D), logCSF Albumin in AD patients (E) and patients with AD+T2D (F), logCSF t-tau in AD patients (G) and patients with AD+T2D (H), logCSF p-tau in AD patients (I) and patients with AD+T2D (J). Data was analyzed with Pearson correlation test, where p<0.05 was considered as significant.
(TIF)

S2 Fig Western blot and A133 immunoblotting analysis of Islet amyloid polypeptide (IAPP) in cerebrospinal fluid (CSF) from cognitively healthy individuals (NC) (n = 43), patients with Alzheimer's disease (AD) (n = 29) and patients with AD and type 2 diabetes (AD+T2D) (n = 7)(A-D).
(TIF)

## References

[1] Zlokovic BV. Neurovascular mechanisms of Alzheimer's neurodegeneration. Trends Neurosci. 2005;28(4):202–8. Epub 2005/04/06. 10.1016/j.tins.2005.02.001 . 15808355

[2] Iadecola C. Neurovascular regulation in the normal brain and in Alzheimer's disease. Nat Rev Neurosci. 2004;5(5):347–60. Epub 2004/04/22. 10.1038/nrn1387 [pii]. . 15100718

[3] Kalaria RN. Small vessel disease and Alzheimer's dementia: pathological considerations. Cerebrovasc Dis. 2002;13 Suppl 2:48–52. Epub 2002/03/20. 49150 [pii] 49150. 10.1159/000049150 . 11901243

[4] Janelidze S, Hertze J, Nagga K, Nilsson K, Nilsson C, Swedish Bio FSG, et al Increased blood-brain barrier permeability is associated with dementia and diabetes but not amyloid pathology or APOE genotype. Neurobiol Aging. 2017;51:104–12. Epub 2017/01/07. 10.1016/j.neurobiolaging.2016.11.017 28061383 PMC5754327

[5] Montagne A, Barnes SR, Sweeney MD, Halliday MR, Sagare AP, Zhao Z, et al Blood-brain barrier breakdown in the aging human hippocampus. Neuron. 2015;85(2):296–302. Epub 2015/01/23. 10.1016/j.neuron.2014.12.032 25611508 PMC4350773

[6] Alafuzoff I, Adolfsson R, Bucht G, Winblad B. Albumin and immunoglobulin in plasma and cerebrospinal fluid, and blood-cerebrospinal fluid barrier function in patients with dementia of Alzheimer type and multi-infarct dementia. J Neurol Sci. 1983;60(3):465–72. Epub 1983/08/01. . 6631444 10.1016/0022-510x(83)90157-0

[7] Blennow K, Wallin A, Fredman P, Karlsson I, Gottfries CG, Svennerholm L. Blood-brain barrier disturbance in patients with Alzheimer's disease is related to vascular factors. Acta Neurol Scand. 1990;81(4):323–6. Epub 1990/04/01. . 2360400 10.1111/j.1600-0404.1990.tb01563.x

[8] Craig-Schapiro R, Kuhn M, Xiong C, Pickering EH, Liu J, Misko TP, et al Multiplexed immunoassay panel identifies novel CSF biomarkers for Alzheimer's disease diagnosis and prognosis. PLoS One. 2011;6(4):e18850 Epub 2011/04/29. 10.1371/journal.pone.0018850 21526197 PMC3079734

[9] Biessels GJ, Kappelle LJ. Increased risk of Alzheimer's disease in Type II diabetes: insulin resistance of the brain or insulin-induced amyloid pathology? Biochem Soc Trans. 2005;33(Pt 5):1041–4. Epub 2005/10/26. BST20051041 [pii] 10.1042/BST20051041 . 16246041

[10] Exalto LG, Biessels GJ, Karter AJ, Huang ES, Katon WJ, Minkoff JR, et al Risk score for prediction of 10 year dementia risk in individuals with type 2 diabetes: a cohort study. Lancet Diabetes Endocrinol. 2013;1(3):183–90. Epub 2014/03/14. 10.1016/S2213-8587(13)70048-2 [pii]. . 24622366 PMC4429783

[11] Schultz N, Byman E, Fex M, Wennstrom M. Amylin alters human brain pericyte viability and NG2 expression. J Cereb Blood Flow Metab. 2016 10.1177/0271678X16657093 . 27354094 PMC5453466

[12] Banks WA, Kastin AJ, Maness LM, Huang W, Jaspan JB. Permeability of the blood-brain barrier to amylin. Life Sci. 1995;57(22):1993–2001. Epub 1995/01/01. . 7475950 10.1016/0024-3205(95)02197-q

[13] Jackson K, Barisone GA, Diaz E, Jin LW, DeCarli C, Despa F. Amylin deposition in the brain: A second amyloid in Alzheimer disease? Ann Neurol. 2013;74(4):517–26. Epub 2013/06/25. 10.1002/ana.23956 23794448 PMC3818462

[14] Despa S, Margulies KB, Chen L, Knowlton AA, Havel PJ, Taegtmeyer H, et al Hyperamylinemia contributes to cardiac dysfunction in obesity and diabetes: a study in humans and rats. Circ Res. 2012;110(4):598–608. 10.1161/CIRCRESAHA.111.258285 22275486 PMC3303627

[15] Lutz TA, Mollet A, Rushing PA, Riediger T, Scharrer E. The anorectic effect of a chronic peripheral infusion of amylin is abolished in area postrema/nucleus of the solitary tract (AP/NTS) lesioned rats. Int J Obes Relat Metab Disord. 2001;25(7):1005–11. Epub 2001/07/10. 10.1038/sj.ijo.0801664 . 11443499

[16] Braegger FE, Asarian L, Dahl K, Lutz TA, Boyle CN. The role of the area postrema in the anorectic effects of amylin and salmon calcitonin: behavioral and neuronal phenotyping. Eur J Neurosci. 2014;40(7):3055–66. Epub 2014/07/22. 10.1111/ejn.12672 . 25040689

[17] Zhu H, Wang X, Wallack M, Li H, Carreras I, Dedeoglu A, et al Intraperitoneal injection of the pancreatic peptide amylin potently reduces behavioral impairment and brain amyloid pathology in murine models of Alzheimer's disease. Mol Psychiatry. 2014 Epub 2014/03/13. 10.1038/mp.2014.17 mp201417 [pii]. 24614496 PMC4161670

[18] Adler BL, Yarchoan M, Hwang HM, Louneva N, Blair JA, Palm R, et al Neuroprotective effects of the amylin analogue pramlintide on Alzheimer's disease pathogenesis and cognition. Neurobiol Aging. 2014;35(4):793–801. 10.1016/j.neurobiolaging.2013.10.076 . 24239383

[19] Qiu WQ, Au R, Zhu H, Wallack M, Liebson E, Li H, et al Positive association between plasma amylin and cognition in a homebound elderly population. J Alzheimers Dis. 2014;42(2):555–63. 10.3233/JAD-140210 24898659 PMC4834912

[20] Schultz N, Byman E, Netherlands Brain B, Wennstrom M. Levels of retinal IAPP are altered in Alzheimer's disease patients and correlate with vascular changes and hippocampal IAPP levels. Neurobiol Aging. 2018;69:94–101. Epub 2018/06/05. 10.1016/j.neurobiolaging.2018.05.003 . 29864717

[21] Westermark P, Andersson A, Westermark GT. Islet amyloid polypeptide, islet amyloid, and diabetes mellitus. Physiol Rev. 2011;91(3):795–826. Epub 2011/07/12. 10.1152/physrev.00042.2009 [pii]. . 21742788

[22] Gong W, Liu ZH, Zeng CH, Peng A, Chen HP, Zhou H, et al Amylin deposition in the kidney of patients with diabetic nephropathy. Kidney Int. 2007;72(2):213–8. Epub 2007/05/15. 5002305 [pii] 10.1038/sj.ki.5002305 . 17495860

[23] Oskarsson ME, Paulsson JF, Schultz SW, Ingelsson M, Westermark P, Westermark GT. In vivo seeding and cross-seeding of localized amyloidosis: a molecular link between type 2 diabetes and Alzheimer disease. Am J Pathol. 2015;185(3):834–46. 10.1016/j.ajpath.2014.11.016 . 25700985

[24] Fawver JN, Ghiwot Y, Koola C, Carrera W, Rodriguez-Rivera J, Hernandez C, et al Islet amyloid polypeptide (IAPP): a second amyloid in Alzheimer's disease. Curr Alzheimer Res. 2014;11(10):928–40. Epub 2014/11/12. . 25387341 10.2174/1567205011666141107124538

[25] Blennow K, Hampel H, Weiner M, Zetterberg H. Cerebrospinal fluid and plasma biomarkers in Alzheimer disease. Nat Rev Neurol. 2010;6(3):131–44. Epub 2010/02/17. 10.1038/nrneurol.2010.4 . 20157306

[26] Olsson B, Lautner R, Andreasson U, Ohrfelt A, Portelius E, Bjerke M, et al CSF and blood biomarkers for the diagnosis of Alzheimer's disease: a systematic review and meta-analysis. Lancet Neurol. 2016;15(7):673–84. Epub 2016/04/14. 10.1016/S1474-4422(16)00070-3 . 27068280

[27] Schultz N, Byman E, Fex M, Wennstrom M. Amylin alters human brain pericyte viability and NG2 expression. J Cereb Blood Flow Metab. 2017;37(4):1470–82. Epub 2016/06/30. 10.1177/0271678X16657093 27354094 PMC5453466

[28] Davson H. Review lecture. The blood-brain barrier. J Physiol. 1976;255(1):1–28. Epub 1976/02/01. 10.1113/jphysiol.1976.sp011267 1255511 PMC1309232

[29] Daneman R. The blood-brain barrier in health and disease. Ann Neurol. 2012;72(5):648–72. Epub 2013/01/03. 10.1002/ana.23648 . 23280789

[30] Biere AL, Ostaszewski B, Stimson ER, Hyman BT, Maggio JE, Selkoe DJ. Amyloid beta-peptide is transported on lipoproteins and albumin in human plasma. J Biol Chem. 1996;271(51):32916–22. Epub 1996/12/20. 10.1074/jbc.271.51.32916 . 8955133

[31] Gotz J, Lim YA, Eckert A. Lessons from two prevalent amyloidoses-what amylin and Abeta have in common. Front Aging Neurosci. 2013;5:38 10.3389/fnagi.2013.00038 23964237 PMC3737661

[32] Yamamoto K, Shimada H, Koh H, Ataka S, Miki T. Serum levels of albumin-amyloid beta complexes are decreased in Alzheimer's disease. Geriatr Gerontol Int. 2014;14(3):716–23. Epub 2013/09/12. 10.1111/ggi.12147 . 24020590

[33] Zhu H, Xue X, Wang E, Wallack M, Na H, Hooker JM, et al Amylin receptor ligands reduce the pathological cascade of Alzheimer's disease. Neuropharmacology. 2017;119:170–81. Epub 2017/04/02. 10.1016/j.neuropharm.2017.03.030 . 28363773 PMC6511375

