# Supplementary figures and images for: Levels of islet amyloid polypeptide in cerebrospinal fluid and plasma from patients with Alzheimer’s disease
Source: PLoS One. 2019 Jun 17;14(6):e0218561. doi: 10.1371/journal.pone.0218561 (PMC6576764; doi:10.1371/journal.pone.0218561)

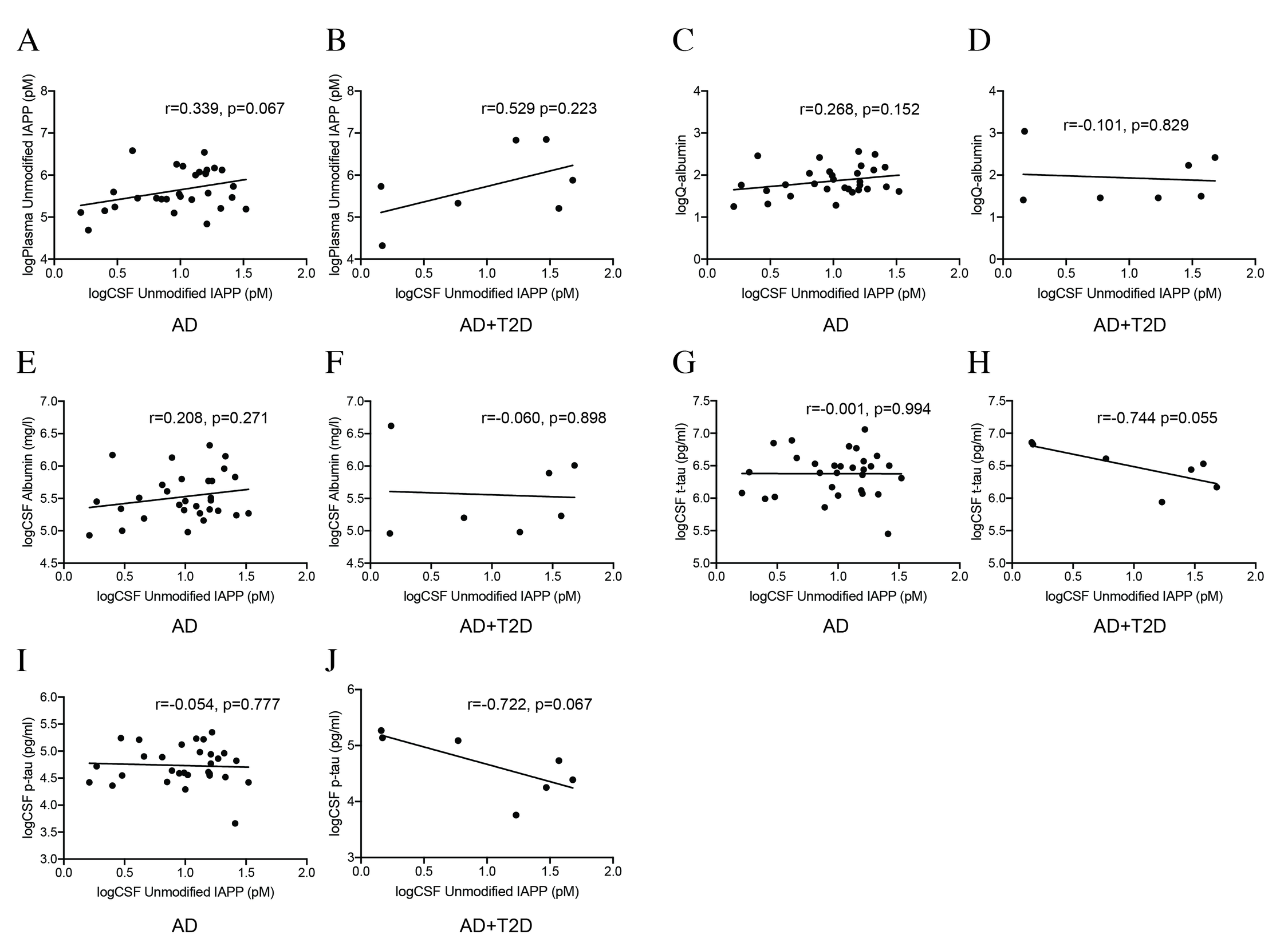

Supplement: S1 Fig — Logarithmic transformed values (log) of CSF unmodified islet amyloid polypeptide (IAPP) where not significantly correlated with logPlasma unmodified IAPP in patients with Alzheimer's disease (AD) (A) and patients with AD and type 2 diabetes (AD+T2D) (B), logQ-albumin in AD patients (C) and patients with AD+T2D (D), logCSF Albumin in AD patients (E) and patients with AD+T2D (F), logCSF t-tau in AD patients (G) and patients with AD+T2D (H), logCSF p-tau in AD patients (I) and patients with AD+T2D (J). Data was analyzed with Pearson correlation test, where p<0.05 was considered as significant. (TIF) [file pone.0218561.s001.tif]

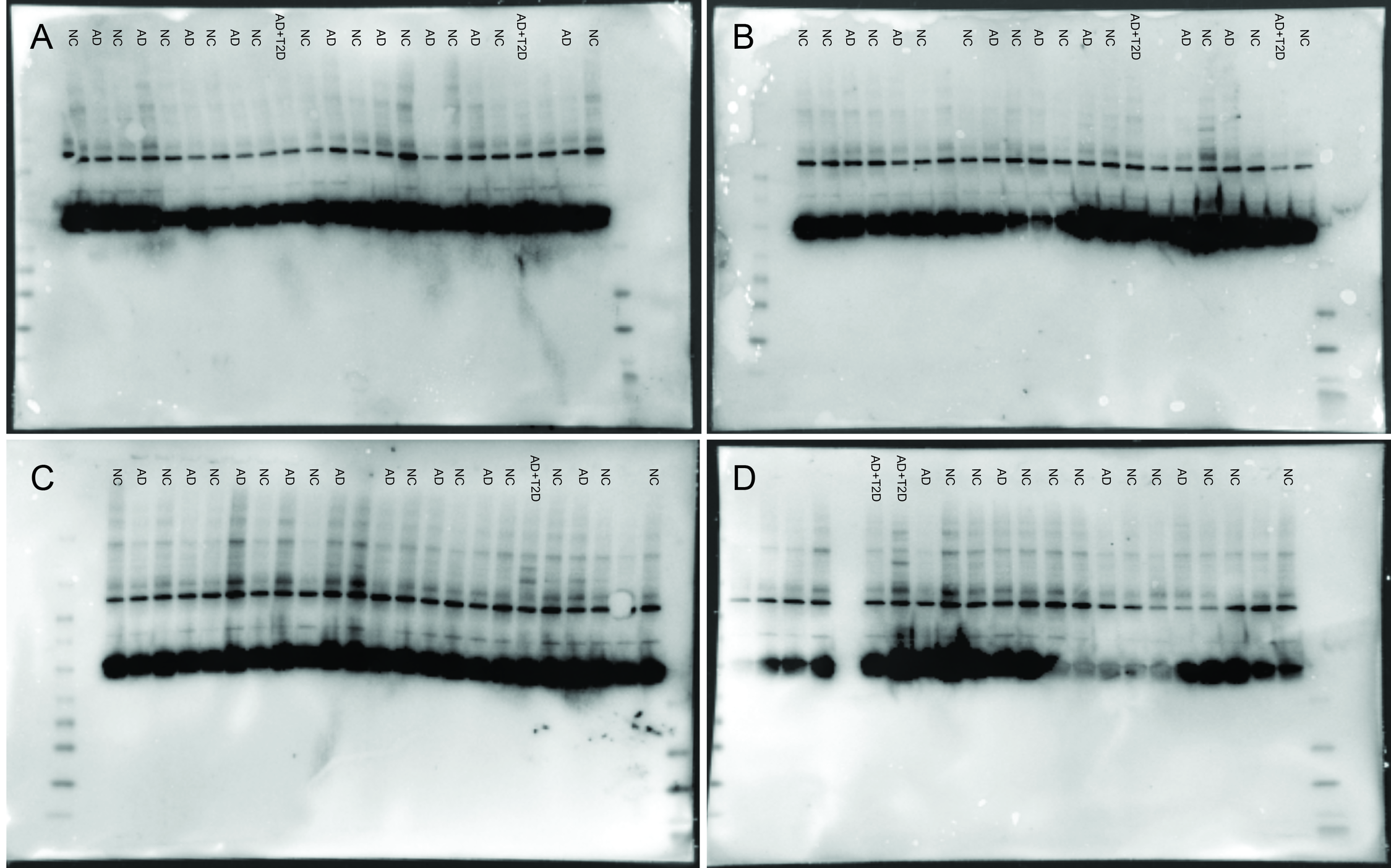

Supplement: S2 Fig — Western blot and A133 immunoblotting analysis of Islet amyloid polypeptide (IAPP) in cerebrospinal fluid (CSF) from cognitively healthy individuals (NC) (n = 43), patients with Alzheimer's disease (AD) (n = 29) and patients with AD and type 2 diabetes (AD+T2D) (n = 7)(A-D). (TIF) [file pone.0218561.s002.tif]
